# Public Service and Public Happiness: Inferences From Big Weibo Datasets for 31 Chinese Provincial Governments

**DOI:** 10.3389/fdata.2022.833703

**Published:** 2022-04-07

**Authors:** Yi Zhou Ding

**Affiliations:** School of Public Affairs, Xiamen University, Xiamen, China

**Keywords:** public service, public happiness, four happiness dimensions, Objective Reality, Subjective Reality, Inter-Subjective Reality, Virtual Reality

## Abstract

The change of public service has usually been considered to affect public happiness. However, since the publication of the Easterlin Paradox, the causal relationship between public service and public happiness has been furiously questioned by public affairs researchers. It has been documented through resolving the four causal factors of public happiness within public administration, new public administration, new public management, and governance that public-service-driven public happiness may be attributed to four happiness dimensions: Objective Reality, Subjective Reality, Inter-Subjective Reality, and Virtual Reality. This article reports the results of significance tests of the relationship between public service and public happiness from analyses of large datasets collected from Weibo systems in 31 Chinese provincial governments from 2010 to 2020. The analyses show that the public service change during this period has not yet led to satisfactory improvement in all four happiness dimensions. Finally, we propose strategies for governments to modify public services to enhance public happiness.

## Introduction

The change of public service^1^ has always been considered to influence public happiness^2^. However, since the publication of the Easterlin Paradox in 1974 (Easterlin, 2016), the proposition “public service acting on public happiness” has been questioned^3^. Through solid empirical studies, various results have been found. The relationship between public service and public happiness has long been undetermined (Dutt and Radcliff, 2009); in other words, the former's change does not guarantee the latter's alternation. The transformation of this strong causal relationship into a weak correlation caused major changes in governance and policy decisions and fueled furious debates among public affairs researchers. However, due to past limits on available technologies and algorithms, we could not grasp the essence of the effect that public service has on public happiness. For example, which aspects of public service influence public happiness? Why does public service affect public happiness inconsistently? How can governments adjust their public service to enhance public happiness? Nevertheless, as new digital tools have become more popular, state-of-the-art measures have been used to answer these questions. This study defines public happiness through a literature review, builds hypotheses to answer the research questions, and uses Python 3.7.3 to study big Weibo datasets of 31 Chinese provincial governments from 2010 to 2020. Then, we discover mediators that exist between public service and public happiness.

## Literature Review

In public administration, researchers refer to public happiness in different ways. Any citizens' feelings stirred by public service can be seen as a change in public happiness. Though it has not been defined, public happiness should be classified as one type of public feeling that can be affected by public service. This means that the contents of public happiness will change when the principal ideas of public service shift in each public management paradigm. Thus, this research reviews the four public management paradigms^4^ to examine the relationship between public service and public happiness, so researchers can know “which parts are missing between the two? how those parts drive research fields to shift? why studying them become important at current?.”

Woodrow Wilson's “The Study of Administration” in 1887 emphasized the modification of administrative efficiency (Box, 2018). During the period of Public Administration, increment in administrative efficiency meant lower service costs and more service benefits (Holzer and Schwester, 2019), it further produces discussions on whether public service can affect public happiness *via* certain mediators. This led researchers to discover one special “reality change” among individuals (Kingsley, 2020) which is mainly linked to the objective aspect of public service, causing citizens' feelings to transform (Lee, 2021). The reality change was called Objective Reality. Due to its function on public happiness, Objective Reality forms one dimension of public happiness and demonstrates that people can gain positive feelings from the outside world. It also drove governments to redesign their public service system and made public servants concentrate more on objective change of public service. They were convinced any visible or tangible change of public service would act on public happiness, for example, some researchers testified public happiness could be enhanced by modifications such as public organizations reinvention (Kuipers et al., 2014) or urban–rural differences adjustment (Mavruk et al., 2021) which would directly affect peoples' feelings through the objective aspect of public service. In practice, citizens' happiness can be altered as long as governments provide services in different ways (Witesman and Walters, 2014). Thus, public happiness may be related to public service *via* Objective Reality. From this perspective, the public administration paradigm is an important phase in the principal development of the idea of public service (Kasdan, 2020) and Objective Reality. However, Objective Reality is not the only happiness dimension. A series of social issues raised in discussions caused scholars to carefully reconsider public service and public happiness.

The new public administration paradigm was born during the Minnowbrook Conference held at Syracuse University in 1968 (Frederickson, 2020). Though that conference did not reach any solid conclusions about modifying public service, it encouraged governments to pay more attention to communication functions that could form certain common sense to enhance public feelings in society (Frederickson, 1996). Moreover, it reminded researchers that there was something still missing between public service and public happiness. The missing part generated by common sense is called Inter-Subjective Reality (Harari, 2015, 2017). For example, citizens are inclined to give positive feedback on the services provided by street-level bureaucrats who are both well-regulated and service-oriented (de Boer, 2020). Actually, Inter-Subjective Reality is one type of common sense formed by communication. Only when governments communicate smoothly with citizens, will there be Inter-Subjective Reality modifications in public service. Otherwise, “citizens automatically and unconsciously associate public sector organizations with inefficiency, inflexibility, and other pejoratives” (Marvel, 2016), causing public happiness failures in society. This is why communication can integrate consciousness and action to some extent (Habermas, 1985). The new public administration paradigm largely focused on the Inter-Subjective Reality dimension. This explains why governments later began to care more about the fairness of public services during civic engagement. Nevertheless, when new research studies emerge, governments still must apply new approaches to increase citizens' happiness (Bok, 2010).

As enterprises were demonstrating better management vitality and increasing more customer satisfaction, governments began to replicate enterprises' experiences and reinvent the public services they provided (Lane, 2002). We called it the new public management movement. The new public management paradigm puts great effort into enhancing citizens' feelings (Osborne and Gaebler, 1993). Though the movement was opposed by some scholars (Denhardt and Denhardt, 2015), it accidentally raised one kind of subjective modification worth reconsideration (Joshanloo, 2019)—governments started to consider whether national service programs could change individuals' subjective wellbeing (Velasco et al., 2019), and “why public organizations were punished more severely by citizens for negative performance information than private organizations” (Van den Bekerom et al., 2021). In fact, those subjective modifications are classified as another reality type, Subjective Reality. Subjective Reality is the third happiness dimension stirred by public service. It implies that public service is able to affect our inner world (e.g., different tones of public servants will bring citizens' different feelings like joy, grief, excitement, fear, anxiety, etc.). However, because it is only one causal factor, Subjective Reality was easily neglected by governments in the past. This is why the new public management movement highlights more citizen-centric public services (Lynn, 2006). With the proper assistance of Subjective Reality, governments can provide satisfactory public service and enhance public happiness more effectively. But even if Objective Reality, Inter-Subjective Reality, and Subjective Reality are integrated, there is still one component missing between public service and public happiness.

Entering the 21st century, the governance paradigm emerged (Ladley, 2019). Combined with the former perspectives, scholars proposed more substantive “public service dominant” approaches (Osborne et al., 2013). To solve sophisticated service issues produced by digitalization, governments applied cutting-edge technologies to conduct data governance (Eryurek et al., 2021). Thus, virtual modification of public services occurred. These virtual modifications originated from what scientists call Virtual Reality. Virtual Reality is the fourth happiness dimension that can influence public feelings *via* digital public services. It also represents the independent electronics world built by information technology and is growing as artificial intelligence (AI) develops (Kaplan, 2015). Once governments collect enough data, they are even capable of predicting human behavior (Barabasi, 2011). That is why some scholars believe that the master algorithm (i.e., an algorithm generated by resolutions of machine learning) can cope with all the practical issues (Domingos, 2018), including modifying public service to fit public happiness. Digital service has no doubt already fused with individuals' feelings, creating one novel happiness dimension. Once governments introduce digital technologies into public service, Virtual Reality will shift public happiness because smart machines are transforming human life forms constantly (Davenport and Kirby, 2016). In smart cities, governments have to modify their services and how they interact with citizens (Ruhlandt, 2018). Only when well-designed technologies are applied, governments can alter human performance efficiently (Shneiderman, 2020) and use Virtual Reality to complete the happiness dimensions.

When researchers commence the establishment of happiness science, it is vital to unlock the relationship between public service and public happiness. Since realities created by public services can influence public happiness, the researcher then uses philosophic methods to summarize Objective Reality, Subjective Reality, Inter-Subjective Reality, and Virtual Reality as four happiness dimensions. After extracting the four happiness dimensions from the four public management paradigms, it is clear why public services can affect public happiness. In other words, different public services will lead to different happiness dimensions, and different happiness dimensions will lead to different levels of public happiness. At this point, the four happiness dimensions become mediators between public service and public happiness. Thus, governments need to predict the potential results before providing public services. Otherwise, the unbalanced weights of the four happiness dimensions will decrease public happiness.

## Research Design

During the period of digitalization, explaining the issue of whether public service affects public happiness requires the application of cutting-edge technology, such as big data and AI. These new technologies can analyze public services and public happiness macroscopically and systematically and offer rational theoretic explanations that fit social reality. Thus, our research design follows the sequence of providing a definition of the concept, stating the research hypotheses, describing the data source, discussing the analysis framework, and selecting the research method in line with other empirical studies.

### Concept Definition

**Figure 1:**
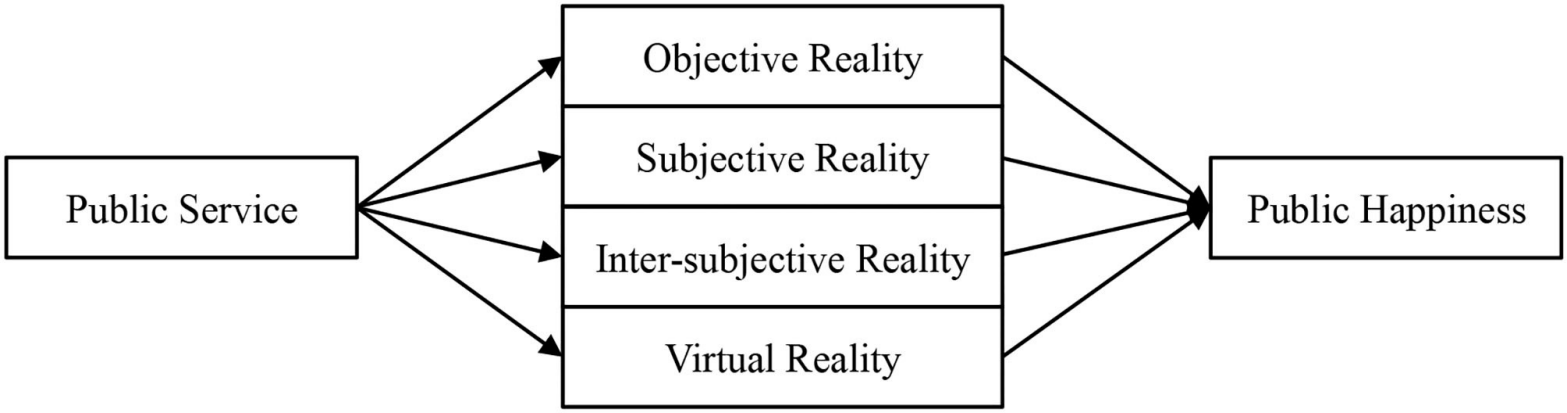
The relationship between public service and public happiness.

Public happiness is hard to understand as citizens expressed their feelings disparately. The concept changes over time and across places and groups, making social sentiment difficult to measure. However, we have observed that the change of public services leads to the alternation in the four happiness dimensions and the alternation will transform public happiness eventually. This implies that a small difference in public service provision can influence Objective Reality, Subjective Reality, Inter-Subjective Reality, and Virtual Reality, causing citizens' feelings to fluctuate. The concept of public happiness thereby becomes a permutation and combination of the four happiness dimensions (Figure 1).

### Research Hypotheses

Indeed, the effects of public services on public happiness are multidimensional. If we want public services to enhance public happiness, then governance has to reach a satisfactory level of improvement in all four happiness dimensions. In reality, however, public service cannot act on all dimensions simultaneously. Thus, to address our research questions, we must verify whether or not public service can affect each happiness dimension in practice. To do so, we test the following hypotheses.

#### Hypothesis 1: Public Service Influences Public Happiness *via* Objective Reality

When governments provide public services, their objective aspects (e.g., landscaping, expressways, school districts, hospital equipment, monitoring system, nursing homes, online businesses, cultural shows, sports facilities, etc.) will affect citizens' feelings accordingly, causing public happiness to fluctuate. Objective Reality functions as a mediator between public service and public happiness. If government modifies objective aspects of public services, the happiness dimension of Objective Reality in citizens' feelings will increase. Thus, they will be satisfied by the provided public service and feel happy.

#### Hypothesis 2: Public Service Influences Public Happiness *via* Subjective Reality

The subjective aspect of public service includes the factors that can affect citizens' inward feelings and change their public happiness (e.g., taking a friendlier tone in public service will give citizens a good impression and add up to their positive feelings). When public servants do not provide citizen-centric public services, citizens may resist them even if the services are high quality in terms of industry standards. This is why citizens' service experiences and satisfaction are becoming increasingly important. To increase public happiness, governments need to change the subjective aspect of public service. Once Subjective Reality functions positively, public happiness will be enhanced.

#### Hypothesis 3: Public Service Influences Public Happiness *via* Inter-Subjective Reality

Citizen participation in governments' activities is required before governments provide certain public services. The more citizens participate, the better social results public service can achieve. This pattern develops through communication among citizens and is the intersubjective aspect of public service (e.g., fairness, public hearings, group sensibility, etc.). When citizens share information about public services, Inter-Subjective Reality is created and public happiness fluctuates. Some citizens might not experience certain public services, but they will obtain information from others and generate positive or negative feelings about it. Under such circumstances, any government interference among citizens will produce unpleasant outcomes, especially official conference interference on citizens' comments on public service.

#### Hypothesis 4: Public Service Influences Public Happiness *via* Virtual Reality

In the age of digitalization, governments introduce digital elements to upgrade traditional public services (e.g., e-payments, smart services, and AI technology) to enhance public happiness. Through this cutting-edge technology, Chinese citizens can handle most of their business from home (in China this is called One-Stop Service), shortening the time needed for its completion and extending people's experiences with virtual aspects of public services. However, Virtual Reality did not exist until the invention of computers and the Internet. This means that Virtual Reality is one new happiness dimension within public happiness. It also proves that public happiness is not the same as it was in the past. If governments do not supplement public service with digital elements, they cannot enhance public happiness at present.

### Data Collection

Pointing to the correlation between public service and public happiness, the researcher collected big Weibo^5^ datasets from 31 Chinese provincial governments and performed analyses on both the posts and public comments on them. This is because Weibo's posts link to public service and Weibo's comments link to public happiness; in other words, we can extract posts and comments from Weibo to map the relationships for further analysis. After analyzing the unstructured data, we acquired information on public services and public happiness from Weibo to answer the research questions.

Based on written and debugged code modules in Python, the researcher crawled Weibo data from 31 Chinese provincial governments between 30 June 2020 and 26 August 2020, obtaining 829,844 blogs and 3,595,105 comments. Due to garbled characters and null rows, among other issues, the researcher immediately cleaned the data, regaining 811,404 blogs and 3,377,687 comments. This information formed the panel dataset from 2010 to 2020. We use 2010 as the starting point because Weibo was established on 29 December 2010 (Sichuan was the first provincial government that used Weibo for government business; Table 1).

**Table 1:** Summary of Weibo data collected from 31 Chinese provincial governments.

**Encoding**	**Province**	**The number of posts**	**The cleaned number of posts**	**The number of times citizens commented on posts**	**The cleaned number of times citizens commented on posts**
1	Anhui	8,872	8,823	4,600	4,377
2	Beijing	66,928	66,588	547,517	516,461
3	Fujian	2,071	2,066	1,489	1,414
4	Gansu	45,151	40,670	82,599	74,233
5	Guangdong	12,700	12,608	0	0
6	Hainan	5,514	5,487	6	5
7	Hebei	4,586	4,555	3,762	3,611
8	Henan	11,647	11,567	10,342	10,021
9	Heilongjiang	3,391	3,363	69,443	68,545
10	Hubei	4,548	4,527	2,503	2,341
11	Hunan	26,814	26,437	22,821	16,728
12	Jilin	52,147	51,211	141,922	135,932
13	Jiangxi	39,115	38,539	89,825	84,098
14	Liaoning	23,879	23,582	3,006	2,905
15	Ningxia	664	663	0	0
16	Qinghai	13,775	13,658	7,803	7,069
17	Shandong	13,018	12,925	22,858	21,603
18	Shanxi	27,519	27,191	44,668	42,970
19	Shaanxi	38,605	38,252	228,754	218,795
20	Shanghai	78,031	76,930	1,048,575	979,971
21	Sichuan	66,769	65,832	321,484	300,742
22	Tianjin	88,361	84,679	439,006	419,694
23	Guizhou	65,121	64,104	123,892	117,158
24	Jiangsu	36,211	35,871	190,014	177,068
25	Tibet	13,828	11,346	10,094	8,979
26	Xinjiang	25,929	25,658	48,941	43,484
27	Inner Mongolia	19,399	19,237	51,909	48,785
28	Yunnan	2,714	2,704	488	457
29	Zhejiang	29,315	29,109	61,918	56,001
30	Guangxi	87	87	0	0
31	Chongqing	3,135	3,135	14,866	14,240
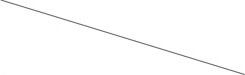	**Total**	829,844	811,404	3,595,105	3,377,687

### Methodological Approach

Because cleaned posts and comments were unstructured data, they could not be used directly in scientific computing. Thus, the researcher used Python to write relative codes to transform the unstructured data into structured data for later analysis. We exploited machine learning and sentiment analysis separately to classify the posts and comments.

#### Machine Learning Analysis of Weibo Posts Dataset

After collecting posts from the Chinese provincial governments, the researcher used machine learning to perform automatic classification. To verify the relationship between public service and public happiness, we sorted Weibo posts into the four groups from the hypotheses: Objective Reality, Subjective Reality, Inter-Subjective Reality, and Virtual Reality (Table 2)^6^.

**Table 2:** Quantification of the four happiness dimensions upon Weibo Blogs.

**Happiness dimensions**	**Problems formulation of weibo blogs**	**Extraction points of weibo blogs**	**Group code**	**Matchable code**
Objective Reality	Did public services affect Objective Reality happiness dimension?	The change of Objective Reality, such as efficacy, effectiveness, efficiency etc.	1	Match 1/Unmatch 0
Subjective Reality	Did public services affect Subjective Reality happiness dimension?	The change of Subjective Reality, such as taking friendlier tones and citizen-centric service etc.	2	Match 1/Unmatch 0
Inter-subjective Reality	Did public services affect Inter-subjective Reality happiness dimension?	The change of Inter-subjective Reality, such as fairness, public hearing, group sensibility etc.	3	Match 1/Unmatch 0
Virtual Reality	Did public services affect Virtual Reality happiness dimension?	The change of Virtual Reality, such as E-payment, smart service, AI technology etc.	4	Match 1/Unmatch 0

After completing this classification scheme, the researcher used a Naive Bayesian Model to complete the codes with Python. The fundamental principle of the Naive Bayesian Model is as follows. Based on the original sample dataset, D = {d_1_, d_2_, d_3_, …, d_n_}, we produced another characteristic dataset, X = {x_1_, x_2_, x_3_, …, x_d_}. Then, we coded the categorical variables as Y = {y_1_, y_2_, y_3_, …, y_m_}. If each variable in {x_1_, x_2_, x_3_, …, x_d_} is random and independent, the prior probability and the posterior probability of Y can be written as P_prior_ = P(Y) and P_post_ = P(Y|X) separately. Then, we produced Equation (1):


(1) $$\begin{array}{l} P\left(Y|X\right)=\frac{P\left(Y\right)P\left(X|Y\right)}{P\left(X\right)} \end{array}$$

Because the variables in the Naive Bayesian Model are independent, we produced Equation (2) according to Equation (1) under the given condition with y class:


(2) $$\begin{array}{l} P\left(X|Y=y\right)=\overset{d}{\underset{i=1}{\prod }}P\left(x_{i}|Y=y\right)\cdots \end{array}$$

We then transformed the posterior probability into Equation (3) through Equations (1) and (2):


(3) $$\begin{array}{l} P_{post}=P\left(Y|X\right)=\frac{P\left(Y\right)\prod_{\text{i}=1}^{d}P\left(x_{i}|Y\right)}{P\left(X\right)}\cdots \end{array}$$

Because the denominator, P(X), does not change, we only needed to compare the posterior probability using the numerators. Thus, we produced Equation (4):


(4) $$\begin{array}{l} P\left(y_{i}|{\text{x}}_{\text{1}},{\text{x}}_{\text{2}},{\text{x}}_{\text{3}},\ldots ,{\text{x}}_{\text{d}}\right)=\frac{P\left(y_{i}\right)\prod_{j=1}^{d}P\left(x_{j}|y_{i}\right)}{\prod_{j=1}^{d}P\left(x_{j}\right)}\cdots \end{array}$$

Once we completed our codes in terms of the model's basic principle, we used random sampling to extract 1% of the data from the 811,404 blogs for training machine learning. According to this process, 80% of the extracted 1% of the data were used for training, and the remaining 20% were used for verification. After ten simulations, the accuracy of the machine learning process reached 80% (Table 3).

**Table 3:** Assessment of machine learning upon Weibo blogs.

**Type**	**Precision**	**Recall**	**F1-score**	**Support**
1	0.85	0.90	0.87	1,793
2	0.59	0.67	0.63	269
3	0.76	0.69	0.72	514
4	0.80	0.30	0.43	152
Accuracy			0.80	2,728
Macro Avg	0.74	0.62	0.65	2,728
Weighted Avg	0.80	0.80	0.80	2,728

#### Sentiment Analysis of Weibo Comments Dataset

Compared with machine learning, sentiment analysis is good at automatically classifying citizens' informal lexis. Thus, we used it to sort 3,377,687 comments into three public happiness categories. Comments containing happy information fell into the positive category (+), those containing unhappy information fell into the negative category (–), and those containing neither happy nor unhappy information fell into the null category (0). After indexes of Weibo comments were established, we implanted Pandas, Numpy Jieba, and other software packages into Python and adopted stop words to complete the relative codes. By doing so, the original text data can be segmented, identified, and classified by sentiment analysis in Python. At that point, the panel dataset of comments from 2010 to 2020 was complete (Table 4).

**Table 4:** Description of happiness classification upon Weibo comments.

**Happiness classification**	**Extraction contents from comments**	**SentiWordNet's contents**	**Group code**
Positive (+)	This type contains the positive sentiment words	Good, like, thumbs-up, great, fantastic etc.	1
Null (0)	This type contains neither the positive or negative sentiment words	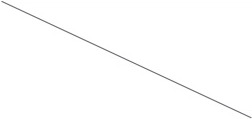	0
Negative (–)	This type contains the negative sentiment words	Not Good, dislike, useless, nonsense etc.	−1

## Empirical Analyses

Using analyses of big data allows us to explore the relationship between public service and public happiness on a large scale. We hypothesized that the change of public services does not always enhance happiness because public services cannot affect some happiness dimensions or affect them negatively. This influences the result of public services and makes it seem like public service and public happiness are unrelated. Thus, solving this puzzle required an advanced methodology to find a reasonable explanation for the weak link between the two variables. Through a series of data processing steps, we used Python codes to transform the Weibo posts and comments into panel data. Then, we analyzed the posts, comments, and post-comment pairs separately to explain their essential properties (Which aspects of public service influence public happiness?), their causes (Why does public service affect public happiness inconsistently?), and their means of influence (How can governments adjust their public services to enhance public happiness?).

### Analyses of Weibo Posts

Since provincial governments in China release most information about public services on Weibo, we can use posts on this platform to inspect the four happiness dimensions produced by public services. After completing the automatic classification through machine learning, we counted the number of posts for each happiness dimension annually. By analyzing processed data from Weibo posts^7^, we can learn which happiness dimensions of public service are perceived by governments to be important.

**Figure 2:**
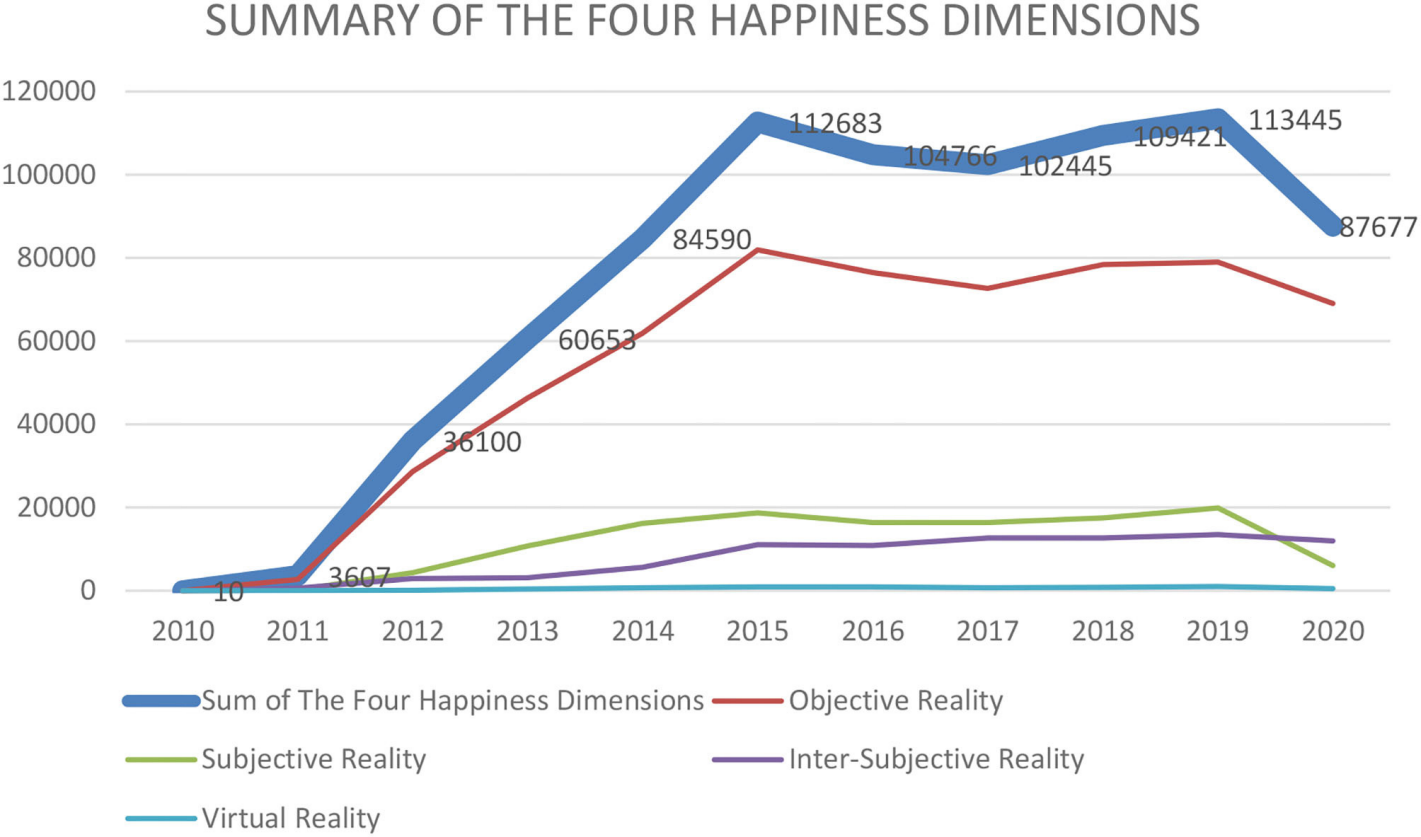
Change of four happiness dimensions over 2010–2020.

Figure 2 shows that change in the happiness dimension of Objective Reality ranks first, change in Subjective Reality ranks second, change in Inter-Subjective Reality ranks third, and change in Virtual Reality ranks fourth^8^. Although the provincial governments understudy continually modified their public services, the objective aspect of public service is what governments modified the most. This means that the provincial governments did not balance the weights of the four happiness dimensions well-enough (Figure 2).

Meanwhile, we further divided the number of posts from 31 provinces into different types, to classify realities generate by public service into four happiness dimensions (i.e., to examine the structures of and differences among the four happiness dimensions in each province). This allowed us to determine how each provincial government should adjust its public services to enhance public happiness.

**Figure 3:**
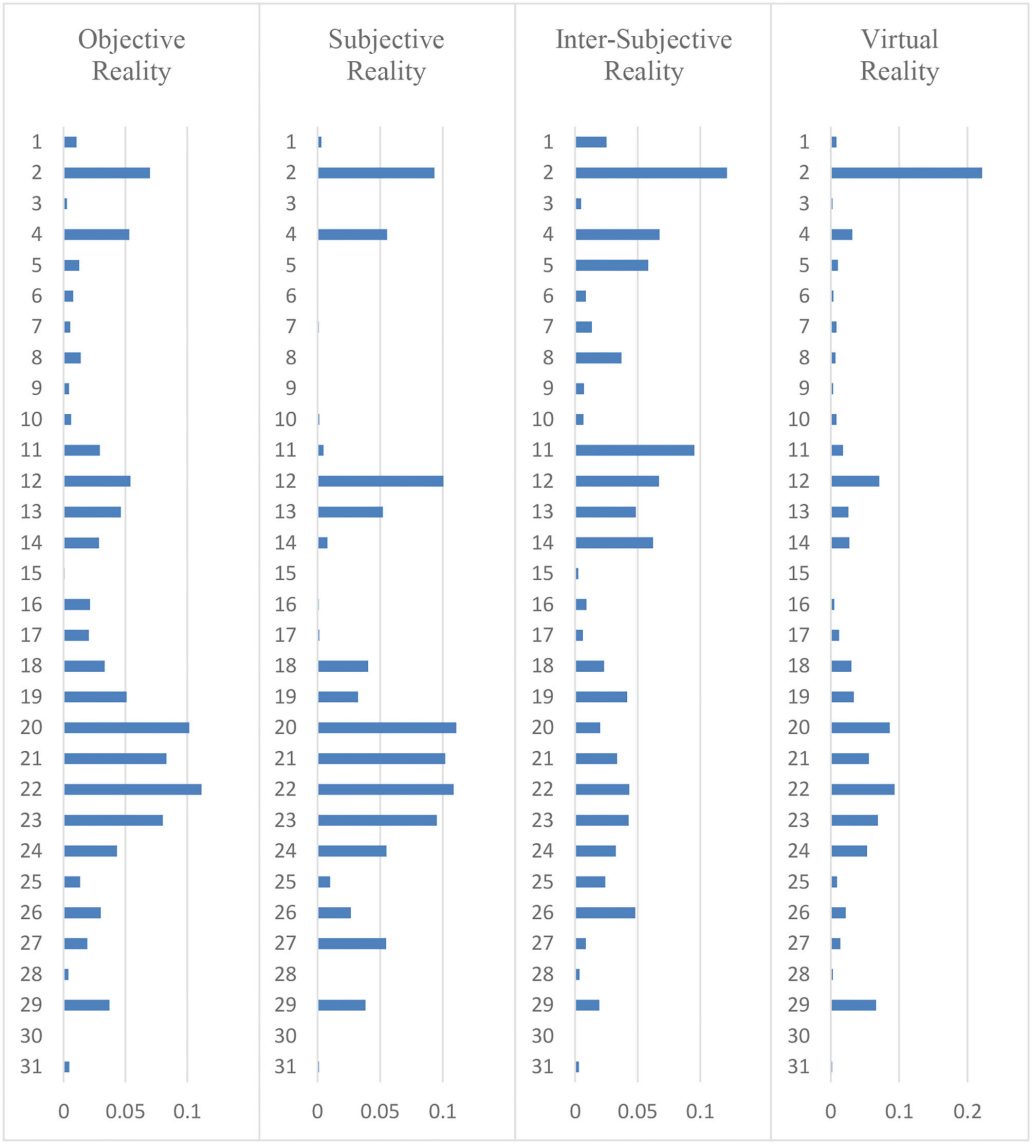
Structure of and difference among the four happiness dimensions from posts datasets.

Figure 3 shows that the top five Objective Reality posts are: Tianjin (22), Shanghai (20), Sichuan (21), Guizhou (23), and Beijing (2). The last five are: Guangxi (30), Ningxia (15), Fujian (3), Yunnan (28), and Heilongjiang (9). The top five cities with Subjective Reality posts are: Shanghai (20), Tianjin (22), Sichuan (21), Jilin (12), and Guizhou (23); the last five are: Guangxi (30), Heilongjiang (9), Ningxia (15), Guangdong (5), and Yunnan (28). The top five cities with Inter-Subjective Reality posts are: Beijing (2), Hunan (11), Gansu (4), Jilin (12), and Liaoning (14); the last five are Guangxi (30), Ningxia (15), Chongqing (31), Yunnan (28), and Fujian (3). Finally, the top five cities for Virtual Reality posts are Beijing (2), Tianjin (22), Shanghai (20), Jilin (12), and Guizhou (23), and the last five are Guangxi (30), Ningxia (15), Chongqing (31), Fujian (3), and Yunnan (28) (Figure 3)^9^.

Though several provincial governments made great efforts to alter local public services, most did not balance the four happiness dimensions. Some provincial governments even had low distributions on all four dimensions because they did not adjust their public services accordingly; examples include Guangxi (30), Ningxia (15), and Heilongjiang (9). Without a theoretical foundation to justify their actions, governments will provide public services aimlessly, causing unbalanced development on the four happiness dimensions. For instance, the provincial government in Anhui (1) mainly focused on changing Objective Reality and Inter-Subjective Reality, so their Weibo posts contained much information about how much they increased basic facilities and how many government conferences they held, but they neglected to discuss changes in Subjective Reality and Virtual Reality. This is likely why their Weibo posts contained less information about how governments provided citizen-centric service (such as taking a friendlier tone) and how many digital elements they added to their public services.

Ultimately, we found that there is a tendency to prioritize the four dimensions in the following order: Objective Reality, Subjective Reality, Inter-Subjective Reality, and Virtual Reality. In other words, most local governments mainly emphasize objective aspects of public services. The best proof of this is that governments prefer to upgrade the hardware of public services (e.g., expanding landscaping areas, expressways, school districts, hospital equipment, monitor units, nursing homes, online businesses, cultural shows, and sports facilities). Meanwhile, they neglect citizens' needs for Subjective Reality, Inter-Subjective Reality, and Virtual Reality. This means that governments have to adjust public servants' working attitudes, communication between government and citizens, and their application of digital technologies. Although some provincial governments (e.g., Guangxi, Ningxia, and others) need to supplement the objective aspect of public services due to low economic development, changes in Objective Reality in other provinces (e.g., Beijing, Shanghai, etc.) are quite extensive. Hence, provincial governments should balance the four happiness dimensions of public service in terms of local economic development. Only when the happiness dimensions are well-balanced, public services can affect public happiness in a sustainable way.

### Analyses of Weibo Comments

Through the analysis of Weibo posts, we discovered that the four happiness dimensions generated by public services are unbalanced. Thus, we also needed to analyze comments on these posts to learn how public happiness changed from 2010 to 2020.

**Figure 4:**
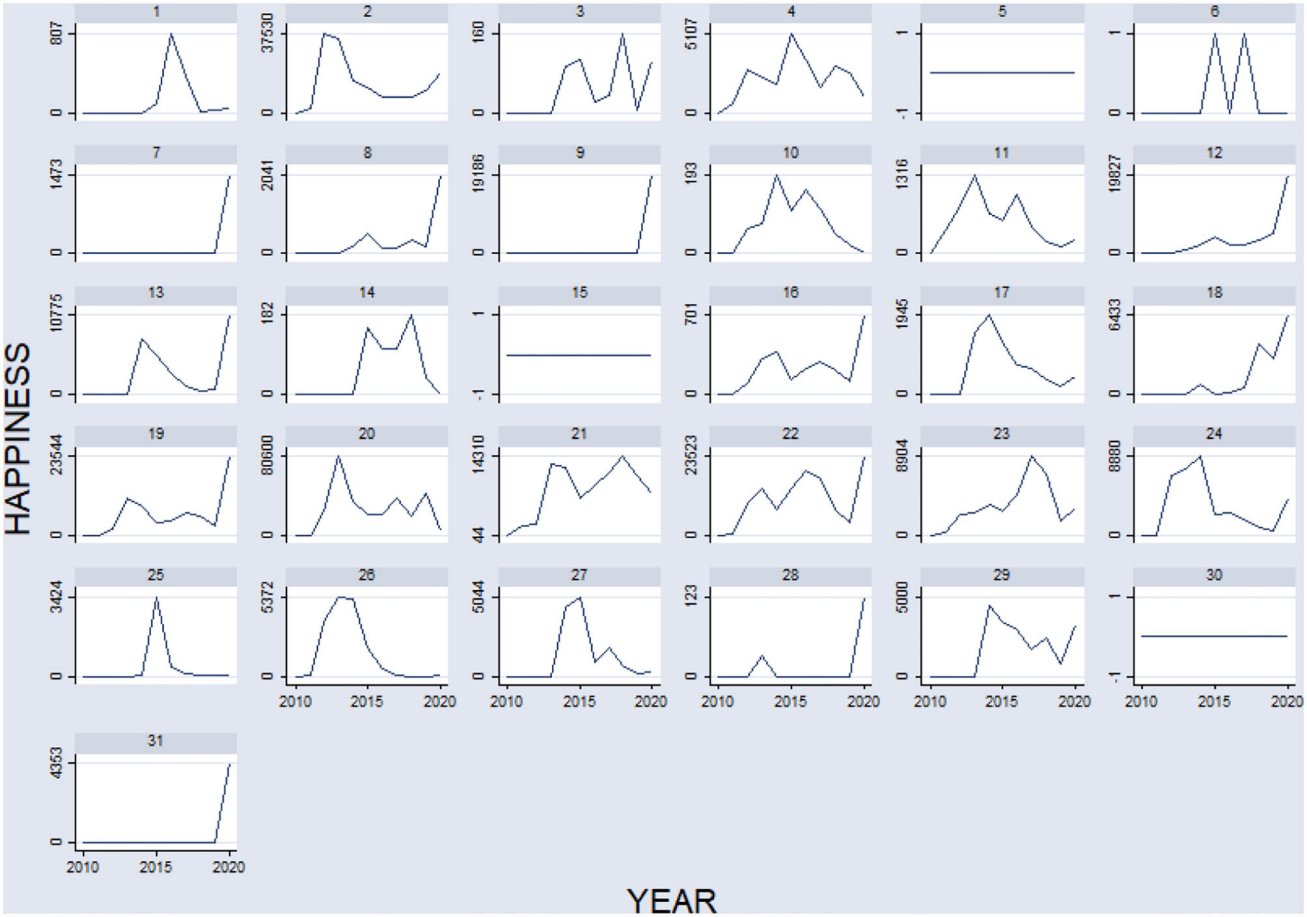
Change of happiness among 31 provinces over 2010–2020.

Using processed comments data formed through sentiment analysis, we classified the 31 provinces into four groups. Group 1 includes those provinces that were unchanged, such as provinces Guangdong (5), Ningxia (15), and Guangxi (30). In Group 1, there was no obvious fluctuation of public happiness among these three provinces from 2010 to 2020^10^. Group 2 represents provinces whose happiness fluctuated over time, such as the provinces of Hainan (6), Tibet (25), and Xinjiang (26). In Group 2, public happiness undulated during some periods but eventually returned to its starting point^11^. Group 3 includes provinces whose happiness increased, such as provinces Anhui (1), Beijing (2), Fujian (3), Hebei (7), Henan (8), Heilongjiang (9), Hunan (11), Jilin (12), Jiangxi (13), Qinghai (16), Shandong (17), Shanxi (18), Shaanxi (19), Tianjin (22), Guizhou (23), Jiangsu (24), Inner Mongolia (27), Yunnan (28), Zhejiang (29), and Chongqing (31). In Group 3, public happiness increased gradually. Though public happiness experienced some changes, it increased conspicuously at the end of the series^12^. Group 4 represents provinces whose happiness decreased, such as provinces Gansu (4), Hubei (10), Liaoning (14), Shanghai (20), and Sichuan (21). In Group 4, although public happiness increased at times, it ultimately declined below its starting point. In sum, the public happiness of 10% of the provinces did not change, and it fluctuated in 10% of the provinces. Public happiness increased overall in 64% of the provinces, and it decreased overall in 16% of the provinces (Figure 4).

After analyzing these changes in public happiness, we concluded that public happiness increased in nearly two-thirds of the provinces. This means that public services can affect public happiness *via* certain methods in China (i.e., *via* Objective Reality, Subjective Reality, Inter-Subjective Reality, and Virtual Reality). Therefore, if we want public happiness to increase, governments must change public services according to the four happiness dimensions. In Figure 4, public happiness fluctuated constantly. This implies that governments did not find the right way to modify public services and keep public happiness stable. Thus, governments should rely on solid public happiness theories while modifying public services. Once the four happiness dimensions are understood, governments are capable of adjusting public services continuously to modify their practices.

Though we analyzed changes in the four happiness dimensions produced by public services and the changes in public happiness in 31 Chinese provinces, a more accurate quantitative analysis is still required. Hence, we connected posts and comments to perform multivariate analyses.

### Multivariate Analyses of Post-comment Pairs

Above, we analyzed Weibo posts and comments individually. Here, we conduct multivariate analyses of post-comment pairs to clarify the precise quantitative relationship between public services and public happiness. Specifically, we explore how public happiness is affected by the four happiness dimensions generated by public services. After reorganizing the panel data in Python, the researcher classified the cleaned number of times citizens commented on posts into three types of posts (+, 0, –), which were used as dependent variables. We then used the cleaned number of posts from the four happiness dimensions as independent variables to establish three models (Table 5).

**Table 5:** Regression models of public happiness from “Blogs-Comments” over 2010–2020.

	**Model 1 happiness (positive)**	**Model 2 happiness (null)**	**Model 3 happiness (negative)**
Happiness of Objective Reality (H1)	2.568^***^ (8.12)	4.651^***^ (7.91)	1.522^***^ (8.00)
Happiness of Subjective Reality (H2)	−1.301 (−1.40)	−1.647 (−0.94)	−1.291^*^ (−2.30)
Happiness of Inter-subjective Reality (H3)	−7.672^***^ (−6.29)	−14.58^***^ (−6.42)	−4.180^***^ (−5.70)
Happiness of Virtual Reality (H4)	30.04 (1.82)	57.62 (1.88)	16.41 (1.65)
cons	256.1 (0.41)	320.0 (0.29)	115.2 (0.31)
N	341	341	341
R-sq	0.46	0.48	0.41
chi2	179.58	187.86	142.44
rho	0.27	0.24	0.26

*t-statistics in parentheses*.

* *p < 0.05*,

^**^p < 0.01,

*** *p < 0.001*.

Comparing the three model types, we discovered that the effects of Objective Reality (H1), Subjective Reality (H2), Inter-Subjective Reality (H3), and Virtual Reality (H4) on public happiness for the three types of posts (+, 0, −) are almost the same. As shown in Table 5, public service only significantly affects Objective Reality and Inter-Subjective Reality. Model 1 shows that Objective Reality (H1) promotes public happiness (β = 2.568, *p* < 0.001). The results indicate that the change of public services in terms of Objective Reality is significant, suggesting that governments made great progress in this area. Subjective Reality (H2), however, does not appear to influence public happiness. The change of public services on this dimension is non-significant, suggesting that governments should change their behavior and attitudes toward citizens. Third, Inter-Subjective Reality (H3) hinders public happiness (β = −7.672, *p* < 0.001). In other words, Inter-Subjective Reality's influence is significant but negative, suggesting the communication between governments and citizens is unpleasant. Finally, Virtual Reality (H4) does not influence public happiness. This means that the change of public services in terms of Virtual Reality is non-significant, suggesting that more public services of this type should be provided. Models 2 and 3 can be analyzed similarly. In brief, changes in public services in China have positive effects on the Objective Reality happiness dimension but no effects on the Subjective Reality or Virtual Reality happiness dimensions, and they have negative effects on the Inter-Subjective Reality happiness dimension.

Lacking the guidance of happiness theory leads to the problem that public services did not affect public happiness. In the age of digitalization, enhancing citizens' public happiness can no longer be accomplished through the traditional provision of public services because this approach is inaccurate and inconsistent. If local governments want to ensure a strong causal relationship between public services and public happiness, then they must balance the four happiness dimensions in practice. By now, most provincial governments in China put great effort into Objective Reality changes without balancing other dimensions, leading the Subjective, Inter-Subjective, and Virtual aspects of public services to fail to reach their full potential. Thus, public happiness increases quite slowly. By examining Weibo of 31 Chinese provincial governments, this research verifies the rationality of four happiness dimensions and guarantees that Objective Reality, Subjective Reality, Inter-Subjective Reality, and Virtual Reality can be balanced. In doing so, public services can consistently affect public happiness.

In conclusion, Hypothesis 1 (public service affects public happiness *via* Objective Reality) and Hypothesis 3 (public service affects public happiness *via* Inter-Subjective Reality) are supported, while Hypothesis 2 (public service affect public happiness *via* Subjective Reality) and Hypothesis 4 (public service affects public happiness *via* Virtual Reality) are not. However, it is important to note that the effect of Hypothesis 1 is positive and the effect of Hypothesis 3 is negative.

## Conclusion

The four happiness dimensions are mediators between public service and public happiness. The structure of and differences among the four happiness dimensions generated by public services can lead to changes in public happiness. If these 31 Chinese provincial governments want public services to influence public happiness, they have to balance Objective Reality, Subjective Reality, Inter-Subjective Reality, and Virtual Reality. Based on the analysis of large datasets collected from Weibo of 31 Chinese provincial governments from 2010 to 2020, we found: most provinces in China modified objective aspects of public services, but other aspects of public services were not changed enough. This is why public services could not promote public happiness in a consistent manner. Therefore, we offer four strategies for modification as follows:

### Strategy 1

Governments should modify objective aspects of public services to enhance public happiness. Weibo of 31 Chinese provincial governments posted an abundant objective change of public services and received strong comments. However, they still need to modify objective aspects of public services according to the situation on the ground. For areas with low development (e.g., Guangxi, Ningxia, Yunnan, etc.), provincial governments should invest more social resources into modifying objective aspects of public services. Meanwhile, high-development areas (e.g., Beijing, Shanghai, Tianjin, etc.) should decrease investments in objective aspects of public services and relocate social resources to different happiness dimensions. Through reexamining the objective aspects of public services, Chinese provincial governments can distribute social resources more precisely.

### Strategy 2

Governments should modify subjective aspects of public services to enhance public happiness. Through these empirical analyses, we found that subjective aspects of public services did not influence public happiness. Most public services provided by provincial governments lack the happiness dimension of Subjective Reality. In fact, Subjective Reality created by public services is vital to promoting public happiness, but it is easy for governments to neglect this facet. To change subjective aspects of public services, governments need to add more citizen-centric elements to their public services to show that they care about citizens' affairs. Furthermore, governments should change the way they provide public services. For instance, public servants often view public services as the government's responsibility instead of theirs, so they inevitably ignore citizens' feelings when they provide public services.

### Strategy 3

Governments should modify intersubjective aspects of public services to enhance public happiness. From the empirical results of the panel data, we found that the change of public services classified as Inter-Subjective Reality did not function positively. This does not imply that the governments did not change public services but that they provided them without proper communication. The Weibo posts provided excessive information about government conferences and changes in public services. However, these top-down government conferences lowered the degree of citizen participation. Thus, the public services that governments provided might not be the public services that citizens wanted. To reverse this trend, provincial governments have to apply more bottom-up methods for public service provision. If governments cannot handle Inter-Subjective Reality, the change of public services will not result in the enhancement of public happiness.

### Strategy 4

Governments should modify virtual aspects of public services to enhance public happiness. Specifically, Chinese provincial governments need to add more digital elements to virtual public services. In Weibo, we found that the number of posts concerning high-tech public services is low. Though governments utilized digital technologies to renovate public services, Virtual Reality is not fully realized in most areas. As the new happiness dimension, the effect of Virtual Reality on public happiness might be tenuous now, but it will be significant in the future. For instance, the rise of the metaverse causes digitalization of public services to develop rapidly, and public happiness is changing even faster. If this happiness dimension is missed, governments will lack an important connection between public services and public happiness. Digital technologies are shifting our way of life, so Chinese provincial governments should reinvent virtual aspects of public services to enhance public happiness.

## Data Availability Statement

The raw data supporting the conclusions of this article will be made available by the authors, without undue reservation.

## Ethics Statement

All procedures performed in studies involving human participants were in accordance with the ethical standards of the institutional and/or national research committee and with the 1964 Declaration of Helsinki and its later amendments or comparable ethical standards.

## Author Contributions

The author confirms being the sole contributor of this work and has approved it for publication.

## Funding

This study was supported by the National Social Science Fund of China (20ZDA108).

## Conflict of Interest

The author declares that the research was conducted in the absence of any commercial or financial relationships that could be construed as a potential conflict of interest.

## Publisher's Note

All claims expressed in this article are solely those of the authors and do not necessarily represent those of their affiliated organizations, or those of the publisher, the editors and the reviewers. Any product that may be evaluated in this article, or claim that may be made by its manufacturer, is not guaranteed or endorsed by the publisher.

## Footnotes

^1^Any good or service provided by government can be classified as a public service.

^2^Public happiness is included in happiness, but it refers only to public feelings that are affected by public service (e.g., when governments expand service area for public transit or increase spending on road maintenance, citizens will probably feel satisfied).

^3^Though Richard A. Easterlin only analyzed happiness in the field of economic growth (operationalized as per capita income), his work indirectly pushed scholars to analyze happiness in other territories (e.g., on October 22–24, 2006), top happiness scholars gathered at the University of Notre Dame and used their own methods to measure happiness. Moreover, Chinese scholars have initiated more happiness studies since 2012, right after the first World Happiness Report was proposed.

^4^The four public management paradigms refer to the four stages that brought new ideas to public administration practice. The researcher used philosophic methods to explore Objective Reality, Inter-Subjective Reality, Subjective Reality, and Virtual Reality through the four public management paradigms.

^5^Weibo (a Microblog site similar to Twitter but only used in China) refers to the social network platform on which users share short real-time information. Weibo of 31 Chinese provincial governments release posts when local public services are modified (e.g., renovation on environment, traffic, education, medical treatment, security, elderly care, administration, culture, sports, etc.). Any civil society members who have Weibo accounts can comment on the government's posts. In China, smartphones are popular across all age groups, including older, rural farmers. Thus, Weibo is representative of the population across China's 31 provinces.

^6^Weibo posts might simultaneously relate to the four happiness dimensions because of overlapping information, but in most case, language can be interpreted into several meanings by postmodernism. Thus, the researcher sorted the posts according to their main type (e.g., we coded a post that mainly contained elements of Objective Reality information into Group 1).

^7^The web crawler began operating on June 30, 2020. Hence, there only 6 months of data were collected during that time period. That is why the trend for this year dropped slightly in Figure 2.

^8^The division is based on the number of posts.

^9^The size of the bars refers to the percentage of the dimension weights.

^10^We cannot predict happiness in Guangdong, Ningxia, and Guangxi accurately because there were no comments on Weibo of these provincial governments. However, according to a survey, for economic motivation, citizens in Guangdong, Ningxia, and Guangxi probably focus more on self-interest and pay no attention to Weibo of the provincial governments.

^11^Public services in these provinces might trigger certain happiness dimensions accidentally, making public happiness fluctuate. However, without solid reasoning supporting government action, public happiness may drop.

^12^Weibo of provincial governments in Chongqing were active for only half a year. As a result, this type needs to be studied further.
